# Immunological Subsets Characterization in Newly Diagnosed Relapsing–Remitting Multiple Sclerosis

**DOI:** 10.3389/fimmu.2022.819136

**Published:** 2022-02-22

**Authors:** Emanuele D’Amico, Aurora Zanghì, Nunziatina Laura Parrinello, Alessandra Romano, Giuseppe Alberto Palumbo, Clara Grazia Chisari, Simona Toscano, Francesco Di Raimondo, Mario Zappia, Francesco Patti

**Affiliations:** ^1^ Department “G.F. Ingrassia”, University of Catania, Catania, Italy; ^2^ Department of Medical and Surgical Sciences, University of Foggia, Foggia, Italy; ^3^ Medicine Department, Neurology Unit, Sant’Elia Hospital, Caltanisetta, Italy; ^4^ Hematology Unit, AOU “Policlinico San Marco”, Catania, Italy; ^5^ Department of General Surgery and Medical–Surgical Specialties, University of Catania, Catania, Italy

**Keywords:** immunophenotype, multiple sclerosis, myeloid cells, T cells, B cells

## Abstract

**Objectives:**

Using flow cytometry, we characterized myeloid, B, and T cells in patients recently diagnosed with relapsing–remitting multiple sclerosis (RRMS) naive to disease-modifying therapies (DMTs).

**Methods:**

This prospective case–control study was conducted in the tertiary MS center of Catania, Italy. Demographic/clinical data and peripheral bloods were collected from 52 naive patients recently diagnosed with RRMS and sex/age-matched healthy controls (HCs) in a 2:1 ratio. We performed flow cytometry on isolated peripheral blood mononuclear cells to assess immune cell subsets differences between RMMS patients and HCs. We explored the biomarker potential of cell subsets using receiver operating characteristic (ROC) curves and relative area under the curve (AUC) analyses.

**Results:**

Monocytic myeloid-derived suppressor cells (Mo-MDSCs CD14+/HLADR^−/low^) and inflammatory monocytes (CD14+CD16+) displayed higher frequencies in RRMS patients when compared with HCs (p <.05). A lower percentage of B-unswitched memory cells was observed in RRMS patients when compared with HCs (p = .026). T cells had a higher frequency of T-helper CD4+ cells and their subset, CD4+CD161+, in RRMS patients when compared with HCs (p <.001). ROC analyses revealed an AUC >70% for Mo-MDSCs CD14+/HLADR^−/low^ and inflammatory CD14+CD16+, T-helper CD3+CD4+, and T-helper CD4+CD161+.

**Conclusions:**

Patients with a recent RRMS diagnosis and naive to DMTs, showed peculiar myeloid, B-, and T-cell immunophenotypes.

## Introduction

Multiple sclerosis (MS) has an unpredictable disease course and clinical manifestation and leads to different levels of inflammatory, demyelinating, and degenerative processes (1, 2). Despite decades of research, few reliable biomarkers have been identified for monitoring the course of MS and treatment responses (3). Currently, using biological markers, it is impossible to predict which MS patients suffer a more severe disease course (4).

Abnormal immune responses are facilitated by the trafficking of peripherally activated immune cells into the central nervous system (CNS). As major drivers of inflammatory disease activity during relapses, these conditions can be targeted by immune-targeting therapies (5, 6).

The characterization of immune cell phenotypes at the time of MS diagnosis, when patients are treatment naive, is poorly understood. Limited data are available on changes in immune cell subsets in peripheral blood mononuclear cells during MS, especially for relapsing–remitting MS (RRMS) (7, 8). In autoimmune diseases and cancer, immune dysfunction is associated with expansion of myeloid-derived suppressor cells (MDSCs), as consequence of emergence hematopoiesis occurring in response to cytokine burden (9, 10). In peripheral lymphoid and tissues, blood stream and spleen, MDSCs act as immune response suppressors, functioning directly or indirectly through the induction of regulatory T cells (11, 12). In humans, MDSCs can be distinguished into monocytic (Mo-MDSC, monocytes and macrophages precursors) and granulocytic (G-MDSC, neutrophils precursors) subtypes (13–15), based on their morphology, immunophenotyping, and functional profiling (16, 17).

Substantial evidence now shows that monocytes and macrophages are prominent myeloid cell types during early disease stages and mediate both pro- and anti-inflammatory responses (18–20). The two distinct monocyte subsets in blood and tissue are characterized by the differential expression of surface and/or secreted antigens and cytokines (13). CD14 and CD16 are both used to distinguish classical (CD14++CD16−) and non-classical (CD14+CD16++, pro-inflammatory) monocyte subsets (13).

In patients with MS, monocytes in the peripheral blood display activation characteristics during disease activity periods (13). However, it is unclear how homeostasis influences pro- and anti-inflammatory monocyte populations in replenishing macrophages at action sites (21, 22).

In this study, we characterized myeloid, B-, and T-cell populations using flow cytometry. We recruited RRMS patients at the time of diagnosis and compared their flow cytometric data with matched healthy controls (HCs). RRMS patients were naive to disease-modifying therapies (DMTs).

## Methods

### Study Design and Settings

This prospective case–control study was conducted at the tertiary MS Center of University of Catania, Italy. Patients were consecutively admitted between October 2020 and July 2021. We included all patients who received a confirmed RRMS diagnosis and who were naive to DMTs.

**Figure 1:**
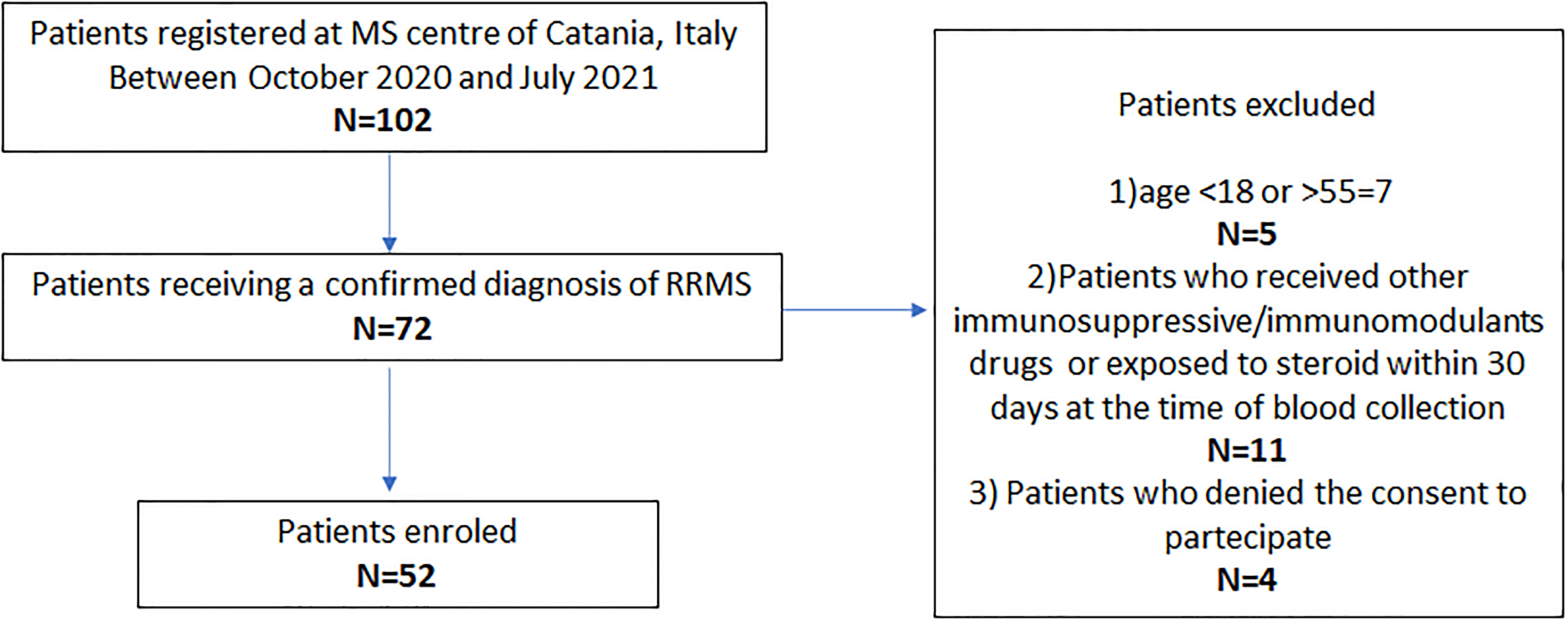
Study flow chart. DMTs, disease-modifying therapies; MS, multiple sclerosis; RRMS, relapsing–remitting MS.

Exclusion criteria were (1) age <18 and >55 years, (2) patients receiving other immunosuppressive/immunomodulant drugs for other diseases or exposed to steroids within 30 days of initial blood collection, and (3) patients who did not consent to participate (**Figure 1**).

All patients underwent clinical and radiological evaluations, and blood and cerebrospinal fluid (CSF) samples were collected at baseline. HCs blood samples were provided by donors from the Hematology Centre of Catania, Catania, Italy. They were randomly age and sex matched to the patients group, in a 2:1 ratio.

### Clinical Assessment and Neuroimaging

Demographic and clinical data were retrieved from a database, iMed^©^ software (iMed^©^, Merck Serono SA, Geneva). These data included (a) demographic data (age and gender), (b) clinical data [disease duration, disability assessed by the Expanded Disability Status Scale (EDSS) and number of relapses in the year before diagnosis], and (c) magnetic resonance imaging (MRI) data (number of brain and spinal cord lesions in T2 weighted and T1 gadolinium-weighted sequences). A cerebral MRI within 6 months of a confirmed diagnosis was considered as baseline MRI. All MRI scans were obtained using the 1.5-Tesla MRI.

CSF was collected by lumbar puncture when the RMMS diagnosis was confirmed. The presence of oligoclonal bands (OB) in the CSF and Link Index [this indicated intrathecal IgG synthesis >0.7 (or the defined laboratory value)] (23, 24).

### Blood Sample Collection and Flow Cytometry

Bloods were collected as part of routine clinical practice. Whole peripheral blood was collected in ethylenediaminetetraacetic acid (EDTA) tubes between 9:00 am and 10:30 am and processed within 2 h. Flow cytometry acquisition was performed using a NAVIOS instrument (Beckman Coulter). For each sample, 100,000 events were acquired.

To evaluate myeloid and T-lymphocyte cells, approximately 1 × 10^6^ cells were labeled with the appropriate monoclonal antibody volume and incubated in the dark at room temperature for 20 min. After staining, cells were lysed in 2 ml 1× ammonium chloride solution and incubated for 10 min at room temperature in the dark. Cells were centrifuged for 5 min at 540 *g*, the supernatant discarded, and 2 ml phosphate buffered saline (PBS) added (these steps were repeated twice). Finally, cells were resuspended in 200 μl PBS and 100,000 events acquired by flow cytometry. To evaluate B-cell populations, a Dura Clone IM B Cell kit was used following manufacturer’s instructions (25).

### Monoclonal Antibodies

To characterize myeloid cell subsets, a panel of nine monoclonal antibodies was used: CD15-FITC (80H5), CD14-PE (RMO52), CD64-ECD (22), CD16-PC5 (3G8), PD-L1-PC7 (PD-L1.3.1), CD33-APC (D3HL60.251), CD38-A750 (LS198-4-3), HLA-DR-PB (Immu-357), and CD45-KO (J33).

To characterize T-cell subsets, a panel of nine monoclonal antibodies was used: PD1-FITC (PD1.3.1.3), CD127-PE (R34.34), CD3-ECD (UCHT1), CD8 FITC (B9.11), CD25-PC5 (B1.49.9), CD4-PC7 (SFCI12T4D11), CD161-ALEXA750 (191B8), CD45RA-PB (2H4LDH11LDB9), and CD45-KO (J33).

To phenotypically characterize B-cell subsets, we used the Dura Clone IM B Cell kit (Beckman Coulter) and a panel of eight monoclonal antibodies: IgD (FITC), CD21 (PE), CD19 (ECD), CD27 (PC7), CD24 (APC), CD38 (APC-750), IgM (Pacific Blue), and CD45 (Krome Orange), according to manufacturer’s instructions.

### Immunological Cell Subset Definitions

#### Myeloid Cells

Cells considered myeloid counterparts were Mo-MDSCs CD14+/HLADR^−/low^, and inflammatory monocytes, CD14+ CD16+. Cells expressing CD15+/CD33+/CD14-/HLADR^−/low^ were defined as G-MDSCs.

#### Lymphocytes

We identified CD16+CD56+ CD3-(natural killer). Among CD3+T cells, we identified CD3+CD4+ (T-helper) and CD3+CD8+ (T-cytotoxic) cells.

Among the T-helper subset, we distinguished the following subpopulations: CD4+CD25+CD127^low^ (T-regulatory cells), T-CD4+CD161+, and CD4+CD45RA+ (T-naive) cells.

After gating for CD19-positive cells, the differential expression of IgM, IgD, CD38, and CD27 permitted identification of the following B-subpopulations: CD19+CD27-IgD+(B-naive), CD19+CD27+CD38-IgM-IgD-(switched B memory), and CD19+CD27+CD38-IgM+IgD+(unswitched B memory) cells.

### Study outcomes

The primary study outcome was the flow cytometric characterization of myeloid, T-, and B-cell subsets in the peripheral blood of newly diagnosed RRMS patients, naive to DMTs. We also assessed if any of these cell subsets could act as disease biomarkers.

In the supplementary section, we collected preliminary clinical and radiological data along with available follow-up after the beginning of first DMT (**Appendix e1**).

### Statistical Analyses

Data were represented as counts (proportions) for categorical variables and mean (standard deviation, SD) or median (interquartile range, IQR) for continuous variables. Chi-square and Fisher exact tests were used to compare categorical variables. Either t-tests or Mann–Whitney U tests were performed to compare lymphocyte subsets depending on Kolmogorov–Smirnov normality test.

A logistic regression analysis with receiver operating characteristic (ROC) curve outputs was performed for each parameter contribution. ROC curves and area under the curve (AUC) were used to measure their respective discriminatory powers, as in previous studies (13). When the discriminatory power was >70%, subsets were considered fair disease markers according to Xia et al. (26).

Correlation analyses were done between significant different cell subsets and clinical/radiological baseline measures (Pearson coefficients and partial Pearson coefficients, age and sex-adjusted, for continuous variables). To examine associations with dichotomic measures (OB), V-Cramer was calculated.

The primary cross-sectional analysis involved the identification of correlations between significantly different cell subsets and baseline EDSS. Secondarily, cross-sectional correlations with the number of relapses in the year prior to diagnosis, the number of brain MRI lesions on T2 weighted and T1 Gadolinium-weighted sequences in the year before diagnosis, and link index at CSF were investigated. Association measures were calculated for OB.

For supplementary analyses methods, see **Appendix e1**.

The model with best inferential properties was chosen in accordance with Akaike information criterion (27).

A p-value ≤0.05 was considered statistically significant. Analyses were performed using SPSS 21.0 (IBM, Armonk, NY, USA).

### Protocol Approval, Registration, and Patient Consent

The study protocol was approved by the Scientific Committee, Comitato Etico Catania I (PO/898/2021). Each participant signed an informed consent sheet prior to participating. This study does not contain any participant identifiers.

### Data Availability

Anonymized data will be shared with qualified investigators, upon reasonable request, for the sole purpose of replicating procedures.

## Results

Patient and HCs demographics, clinical and radiological characteristics, and cell subsets comparisons between groups are shown (**Table 1**).

**Table 1:** Baseline cohort characteristics and cell subsets.

	RRMS	HC	p-value*
	(n = 52)	(n = 26)	
Female n (%)	35 (67.3)	18 (70)	.120
Age (year), (mean ± SD)	35.7 ± 11.1	34.1 ± 12.8	.671
Relapses in the year before diagnosis (mean ± SD)	1.8 ± 0.8	**–**	**–**
Baseline EDSS (median, IQR)	1.5 (1.0-2.5)	**–**	**–**
No. of brain MRI lesions on T2 weighted sequences (mean ± SD)	25.5 ± 21.1	**–**	
No. of brain MRI lesions on T1 gadolinium weighted sequences (mean ± SD)	1.4 ± 4.9	**–**	
**Myeloid cells** % (SD)			
G-MDSC	59.2 (7.2)	58.7 (4.7)	.748
CD15+/CD33+/CD14-/HLADR^−/low^			
Mo-MDSC	9.3 (3.2)	7.5 (1.8)	.022
CD14+/HLADR^−/low^			
Inflammatory monocytes	12.5 (6.1)	5.6 (2.8)	.002
CD14+/CD16+			
**Lymphocytes,** % (SD)			
Natural Killer CD16+CD56+CD3-	11.1 (5.2)	12.9 (4.7)	.096
T CD3+	74.8 (7.1)	71.9 (6.4)	.107
T-helper CD3+CD4+	46.6 (6.8)	41.2 (4.9)	.001
T-cytotoxic CD3+CD8+	28.1 (8.1)	30.3 (3.9)	.197
CD4+/CD8+ (ratio, mean ± SD)	1.8 ± 0.8	1.4 ± 0.2	.005
T-helper CD4+CD45RA+ (naive)	37.7 (11.6)	36.3 (8.1)	.794
T-helper CD4+CD161+	8.6 (6.1)	3.5 (1.9)	.002
T-reg CD4+CD25+CD127low/−	7.0 (2.6)	6.5 (1.5)	.651
B cells CD19+	11.4 (4.4)	9.5 (4.0)	.162
B-naive, CD19+CD27-IgD+	60.4 (10.1)	60.9 (4.6)	.151
Switched B memory CD19+CD27+CD38-IgM-IgD−	18.7 (7.4)	19.3 (3.5)	.201
Unswitched B memory CD19+CD27+CD38-IgM+IgD+	10.8 (4.6)	13.2 (3.3)	.026

*Chi-square and Fisher exact tests were used to compare categorical variables. Lymphocyte subsets were compared between groups and either t-tests or Mann–Whitney U tests were performed depending on the Kolmogorov–Smirnov normality test results. EDSS, Expanded Disability Status Scale; MRI, magnetic resonance imaging; No., number; SD, standard deviation.

**Figure 2:**
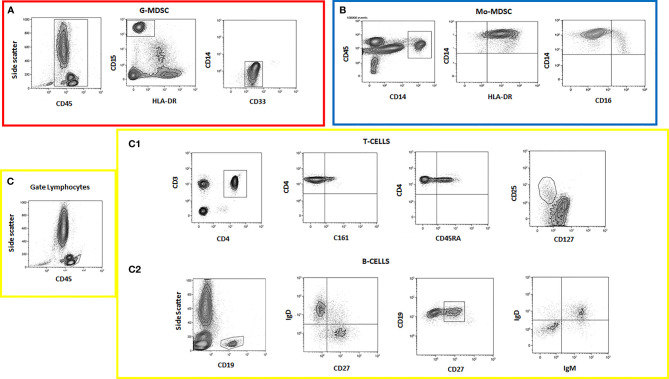
Flow cytometry data showing different cell subset analyses. **(A)** G-MDSC panel; **(B)** Mo-MDSC panel; **(C)** lymphocyte panel; (C1) T cells; (C2) B cells.

The gating strategy identifying different cell subsets is shown (**Figures 2A–C**).

**Figure 3:**
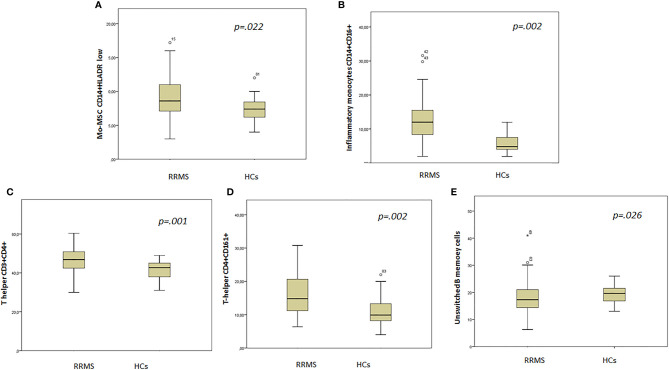
Box plot showing statistically different cell subsets between groups. **(A)** Mo-MDSC CD14+HLADR low; **(B)** inflammatory monocytes CD14+CD16+; **(C)** T helper CD3+ CD4+; **(D)** T helper CD4+CD161+; **(E)** unswitched B memory cells.

Myeloid cell analysis showed that patients had higher Mo-MDSCs CD14+/HLADR^−/low^ (9.3%, SD 3.2% vs. 7.5%, SD 1.8%, p = .022) and inflammatory monocytes, CD14+/CD16+ (12.5%, SD 5.6% vs. 5.6%, SD 2.8%, p = .002) when compared with HCs (**Figures 3A, B** **)**.

T-cell subset analysis revealed higher T helper cells CD3+CD4+ (46.6%, SD 6.8% vs. 41.2%, SD 4.9%, p = .001) in patients when compared with HCs (**Figure 3C**). In addition, the CD4+/CD8+ ratio was higher in patients (1.8, SD 0.8 vs. 1.4, SD 0.2, p = .001) than in HCs.

The T-helper CD4+CD161+ cell subset was higher in patients than in HCs (16.1%, SD 6.2% vs. 10.9%, SD 4.6%, p = .002) (**Figure 3D**).

B-cell analysis revealed lower unswitched B memory cells in patients when compared with HCs (10.1%, SD 4.5% vs. 13.2%, SD 3.1%, p = .026) (**Figure 3E**).

### ROC Curves

**Figure 4:**
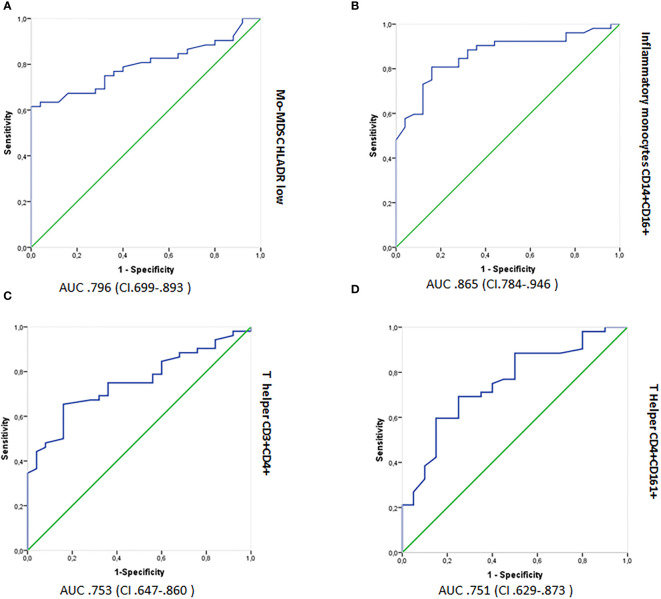
Logistic regression analyses showing ROC outputs for patients with RRMS plotted against HCs. AUC, with 95% confidence intervals (CI), is provided for each parameter. The surface expression of parameters in RRMS patients are combined as true positives (n = 52) and plotted against HCs (n = 26) as true negatives. The diagonal dividing the ROC represents random events. A logistic regression analysis of combined parameter results was performed for “all parameters,” parameters with AUC >0.70 and >0.75. ROC, receiver operating characteristic; AUC, Area under the curve; RRMS, relapsing–remitting MS; HCs, healthy controls. **(A)** Mo-MDSCs CD14+/HLADR−/low, **(B)** inflammatory monocytes, CD14+CD16+, **(C)** T helper CD3+CD4+, and **(D)** T helper CD4+CD161+.

ROC curves with AUC were used to measure the discriminatory power of cell subsets as potential biomarkers (**Figures 4A–D**). From the graphs, the discriminatory power of (a) Mo-MDSCs CD14+/HLADR^−/low^, (b) inflammatory monocytes, CD14+CD16+, (c) T helper CD3+CD4+, and (d) T helper CD4+CD161+ populations was >70%; thus, these cell subsets could be considered as disease biomarkers (26).

### Cross-Sectional Analyses

Correlation analyses between cell subsets of interest and first EDSS showed a transition of significance with inflammatory monocytes, CD14+CD16+ (p = .056, rho = .267). When the analysis was corrected for age and sex, no correlations were found (**Table 2** **)**. Other correlation analyses showed no primary correlations, even when corrected for age and sex (**Table 2**).

**Table 2:** Correlation analyses between cell subsets and clinical/radiological parameters at disease onset.

Variables	r*	p-value	r*	p-value
	coefficient unadjusted		coefficient sex/age/adjusted	
**Inflammatory monocytes, CD14+CD16+**				
Baseline EDSS	.267	.056	.130	.362
No. of relapses one year before diagnosis	−.073	.606	−.016	.909
No. of brain MRI lesions on T2 weighted sequences one year before diagnosis	.041	.744	.073	.609
No. of brain MRI lesions on T1-gadolinium weighted sequences 1 year before diagnosis	.122	.387	.143	.208
Link index value	.110	.290	.125	.300
**T helper CD3+CD4+**				
Baseline EDSS	.135	.340	.172	.226
No. of relapses 1 year before diagnosis	.057	.705	.074	.604
No. of brain MRI lesions on T2 weighted sequences 1 year before diagnosis	−.004	.978	.006	.969
No. of brain MRI lesions on T1-gad weighted sequences 1 year before diagnosis	.171	.225	.114	.425
Link index value	.161	.213	.100	.325
**T helper CD4+CD161+**				
Baseline EDSS	.227	.106	−.011	.937
No. of relapses 1 year before diagnosis	−.270	.073	−.247	.081
No. of brain MRI lesions on T2 weighted sequences 1 year before diagnosis	−.035	.803	−.021	.885
No. of brain MRI lesions on T1-gad weighted sequences 1 year before diagnosis	.122	.387	.195	.170
Link index value	.102	.298	.175	.155
**Unswitched B memory cells**				
Baseline EDSS	.127	.136	−.031	.737
No. of relapses 1 year before diagnosis	−.310	.084	−.324	.091
No. of brain MRI lesions on T2 weighted sequences 1 year before diagnosis	−.068	.904	−.061	.775
No. of brain MRI lesions on T2 weighted sequences 1 year before diagnosis	.116	.187	.185	.165
Link index value	.132	.401	.201	.145

*Pearson correlation coefficients and partial Pearson correlation coefficients were age and sex adjusted. EDSS, Expanded Disability Status Scale; MRI, magnetic resonance imaging; No., number.

### Ancillary Outcome: Follow-Up Preliminary Data

Follow-up preliminary data are shown (**Appendix e1** and **Supplementary Table e1**).

## Discussion

RRMS patients naive to DMTs showed a higher percentage of Mo-MDSCs than HCs, with ROC curves confirming Mo-MDSCs and inflammatory monocytes, CD14+CD16+ as potential disease biomarkers. The B counterpart showed a reduced rate of unswitched B memory cells when compared with HCs. Additionally, T CD4+ and T-CD4+CD161+ cells were higher in RRMS patients than in HCs and were confirmed as potential RMMS biomarkers from ROC analyses.

A recent study identified high inflammatory monocytes, CD14+CD16+ in 25 RRMS patients when compared with 20 HCs (8). We showed that inflammatory monocyte CD14+CD16+ counterparts could be considered as potential disease biomarkers when compared with HCs. In addition, based on established nomenclature, we provided strong evidence that this cell expansion was primarily attributable to non-classical monocyte populations. The characterization of monocyte subsets in MS could help clarify disease mechanisms and facilitate therapeutic development to target inflammatory macrophages and other activated immune cells recruited in the CNS from peripheral blood (13). Thus, non-classical monocytes could act as alternatives to T- and B-cells depletion strategies with the increased advantage of leaving the major classical monocyte population untouched and able to react toward new infection or reinfections (12). Alternatively, selective monocyte subset depletion could supplement existing therapies to increase therapeutic efficacy. Studies on other chronic inflammatory diseases have demonstrated the expansion of CD16+ monocyte populations, further highlighting the importance of non-classical monocyte populations during MS inflammation (12, 14, 28). A recent systemic lupus erythematosus study showed that CD16+ monocytes, characterized by different cell-surface marker profiles, were enriched and had critical roles in driving pathogenic T- and B-cell responses in patients with the disease (29). Furthermore, a rheumatoid arthritis study showed that blood monocyte maturation into tissue-infiltrative CD16+ cells before entry into the joint, induced by cytokine spill-over from the inflamed joint, may have contributed to persistent joint inflammation in this disease (30). Thus, monocyte subset characterization in MS could provide important insights on disease mechanisms and generate potential therapeutic targets, as inflammatory macrophages and other activated immune cells in demyelinating lesions in the CNS are recruited from the peripheral blood.

Recent successful MS therapeutic strategies have involved the specific depletion of peripheral blood cell populations, such as B cells, using rituximab, ocrelizumab, and ofatumumab, or preventing activated T cell access into the CNS, using natalizumab (31–36). Similar strategies targeting monocytes or myeloid lineage cells could be potentially used for MS therapy.

We also provided valuable insights on the reduced frequency of unswitched B memory cells in RRMS patients. Research in the MS field has recently focused on B memory cells; however, no specific, unequivocal theories have been proposed (37). Many DMTs, believed to act *via* T-cell inhibition, can also deplete CD19+ and CD27+ memory B cells (38). Unmutated and unswitched (IgM+) B memory cells are typically germinal center independent; thus, they retain the potential for adaptability within the memory B cell pool, thereby maintaining poly-reactivity (38). The reduced unswitched B-cell frequency associated with increased switched B memory counterparts has been described for other autoimmune diseases, such as systemic lupus erythematosus patients before rituximab treatment (28). Patients with prolonged clinical remission experience delays in both switched and unswitched memory B-cell reconstitution (28). A recent study investigating human unswitched B memory cells, despite sharing high phenotypic similarities with switched memory B lymphocytes, potentially showed high stimulation by activated neutrophils in early inflammation (39, 40). Thus, activated neutrophils at early inflammation stages could attract and modulate unswitched B memory cells, specifically inducing their differentiation into plasma cells, thereby creating a unifying concept centered on interactions between cell and innate immunity (39, 40).

We also confirmed the role of T-CD4+ cells in early RMMS stages. In particular, T-subset CD4+CD161+ cells emerged as potential disease biomarkers. CD161+ cells are markers of human memory Th17 cells, a cell subset that exhibits inflammatory activity and represents a major target of MS therapy; Th17 cells are abundant in peripheral blood, CSF, and brain lesions in MS patients, and their inflammatory mediators are increased during disease relapse (41).

Our data encourage further systemic studies to quantify cell subsets and associated cytokines in different diseases.

In terms of study robustness, the strict standardization of flow cytometry assays was essential for the full characterization of immunophenotype in MS patients. Similarly, we conducted this research in the same center using a uniform methodology, and all patients were naive to DMT/steroids at initial blood collection. In contrast, some flow cytometry studies are heterogeneous in nature with patients variably exposed to different immunosuppressant and steroids (42).

Our study had several limitations: (1) a relatively small sample size; (2) the typical intrinsic limits of immune phenotype studies, including normal inter-individual immune system variations relative to time dependent changes and heritable and non-heritable influences from microbial and environmental factors. These factors are typically manageable in small samples with homogeneous populations; however, they can limit the generalizability of results.

Our data should be integrated into long-term, follow-up studies, with power calculated sample sizes. In addition, comparative studies with other non-MS neurological pathologies would be beneficial to identify new individualized and immunophenotype-oriented therapies for RMMS.

## Data Availability Statement

The raw data supporting the conclusions of this article will be made available by the authors, without undue reservation.

## Ethics Statement

The studies involving human participants were reviewed and approved by Comitato Etico Catania I (PO/898/2021). The patients/participants provided their written informed consent to participate in this study.

## Funding

This study received funding from Biogen srl, BGT-11698 and by Research Funding for University of Catania, Italy (Research Plan 2018-2020 PIACERI Linea 2). The funders were not involved in the study design, collection, analysis, interpretation of data, the writing of this article, or the decision to submit it for publication.

## Conflict of Interest

All authors declare that the research was conducted in the absence of any commercial or financial relationships that could be construed as a potential conflict of interest.

## Publisher’s Note

All claims expressed in this article are solely those of the authors and do not necessarily represent those of their affiliated organizations, or those of the publisher, the editors and the reviewers. Any product that may be evaluated in this article, or claim that may be made by its manufacturer, is not guaranteed or endorsed by the publisher.
