# Supplementary material for: Immunological Subsets Characterization in Newly Diagnosed Relapsing–Remitting Multiple Sclerosis
Source: Front Immunol. 2022 Feb 22;13:819136. doi: 10.3389/fimmu.2022.819136 (PMC8902351; doi:10.3389/fimmu.2022.819136)
Supplement: Supplementary file 1 [file DataSheet_1.docx]

**Appendix e1. Longitudinal preliminary analysis along the availabe follow up**

**Methods**

The longitudinal analyses examined the association between rate of cellular subsets statistically different at disease onset and disease activity after the beginning of the first DMT.

A binary logistic regression model was built to explore the effect of demographical, clinical, CSF, radiological and cellular subsets variables on the probability of occurrence of disease activity (expressed as dichotomic 0/1) along the available follow up following the beginning of first DMT.

Disease activity was defined as new or enlarging T2 lesions on brain MRI sequences or new T1-Gadolinium lesions or new clinical relapses. The model with best inferential properties was chosen according to AIC value.

We investigated several variables: patient’s age at inclusion (as a continuous variable), DMTs’line (as dichotomic, 0=first line; 1=second line), baseline EDSS, number of relapses one year before diagnosis, the number of brain MRI lesions on T2 and T1 Gadolinium weighted sequences within 12 months before diagnosis (all as a continuous variable), OB presence (as dichotomic; 0=no; 1=yes), Link-index value and cellular subsets significantly different at previous descriptive analyses.

**Results**

All RRMS patients started a DMT after the diagnosis confirmation and none discontinued treatment prior to the last follow-up. No differences were found about the rate of first and second line DMTs prescribed: 32/52 (61.5%) RRMS patients were on first line DMT, whilst 20/52 (38.5%) were on second line DMT (p=.141).

Along the investigated follow up (median 8.3 months, interquartile range 4.3-11.8), four patients had clinical relapses (all within the first six months on therapy), one patient experienced radiological activity, whilst there was no increase of disability. Additionally, our analyses assessing the relationship between disease activity (after the beginning of first DMT) and cellular subsets demonstrated no associations (Table e1).

**Table e1 Logistic regression model to explore the probability of occurrence of disease activity along the available follow up following the beginning of first DMT.**

|  | **Exp(B)** | **CI (95%)** | **p-value** |
| --- | --- | --- | --- |
| Age | .996 | .801-1.239 | .973 |
| ^1^DMTs’line | 1.109 | .046-26.981 | .949 |
| Baseline EDSS | 1.968 | .413-6.983 | .463 |
| N. of relapses one year before diagnosis | .471 | .022-10.050 | .630 |
| N. of Brain MRI lesions on T2 weighted sequences one year before diagnosis | .745 | .933-1.102 | .609 |
| N. of Brain MRI lesions on T1-gadolinium weighted sequences one year before diagnosis | .437 | .520-4.533 | .208 |
| ^1^OB presence | 1.364 | .065-10.213 | .876 |
| Link-index value | .437 | .350-5.420 | .198 |
| Inflammatory monocytes, CD14+CD16+ | 1.044 | .840-1.297 | .700 |
| T helper CD3+CD4+ | 1.101 | .844-1.437 | .478 |
| T helper CD4+CD161+ | .793 | .506-1.241 | .309 |
| Unswitched B-memory cells | 1.200 | .498-1.980 | .290 |

*CI, confidence interval; EDSS, Expanded Disability Status Scale; MRI, magnetic resonance imaging; N.,number; OB, oligoclonal bands.

^1^for dichotomic variables, the last category was considered as reference.
